# Probing the *VIPR2* Microduplication Linkage to Schizophrenia in Animal and Cellular Models

**DOI:** 10.3389/fnins.2021.717490

**Published:** 2021-07-22

**Authors:** Yukio Ago, Satoshi Asano, Hitoshi Hashimoto, James A. Waschek

**Affiliations:** ^1^Department of Cellular and Molecular Pharmacology, Graduate School of Biomedical and Health Sciences, Hiroshima University, Hiroshima, Japan; ^2^Laboratory of Molecular Neuropharmacology, Graduate School of Pharmaceutical Sciences, Osaka University, Suita, Japan; ^3^Molecular Research Center for Children’s Mental Development, United Graduate School of Child Development, Osaka University, Kanazawa University, Hamamatsu University School of Medicine, Chiba University and University of Fukui, Suita, Japan; ^4^Division of Bioscience, Institute for Datability Science, Osaka University, Suita, Japan; ^5^Open and Transdisciplinary Research Initiatives, Osaka University, Suita, Japan; ^6^Department of Psychiatry and Biobehavioral Sciences, Semel Institute for Neuroscience and Human Behavior, David Geffen School of Medicine, University of California, Los Angeles, Los Angeles, CA, United States

**Keywords:** VPAC2 receptor (*VIPR2*), schizophrenia, psychiatric disorders, neurodevelopment, synaptic plasticity, cognition

## Abstract

Pituitary adenylate cyclase-activating polypeptide (PACAP, gene name *ADCYAP1*) is a multifunctional neuropeptide involved in brain development and synaptic plasticity. With respect to PACAP function, most attention has been given to that mediated by its specific receptor PAC1 (*ADCYAP1R1*). However, PACAP also binds tightly to the high affinity receptors for vasoactive intestinal peptide (VIP, *VIP*), called VPAC1 and VPAC2 (*VIPR1* and *VIPR2*, respectively). Depending on innervation patterns, PACAP can thus interact physiologically with any of these receptors. VPAC2 receptors, the focus of this review, are known to have a pivotal role in regulating circadian rhythms and to affect multiple other processes in the brain, including those involved in fear cognition. Accumulating evidence in human genetics indicates that microduplications at 7q36.3, containing *VIPR2* gene, are linked to schizophrenia and possibly autism spectrum disorder. Although detailed molecular mechanisms have not been fully elucidated, recent studies in animal models suggest that overactivation of the VPAC2 receptor disrupts cortical circuit maturation. The *VIPR2* linkage can thus be potentially explained by inappropriate control of receptor signaling at a time when neural circuits involved in cognition and social behavior are being established. Alternatively, or in addition, VPAC2 receptor overactivity may disrupt ongoing synaptic plasticity during processes of learning and memory. Finally, *in vitro* data indicate that PACAP and VIP have differential activities on the maturation of neurons via their distinct signaling pathways. Thus perturbations in the balance of VPAC2, VPAC1, and PAC1 receptors and their ligands may have important consequences in brain development and plasticity.

## Introduction

Pituitary adenylate cyclase-activating polypeptide (PACAP) and the closely related neuropeptide vasoactive intestinal peptide (VIP), exhibit widespread expression in the central and peripheral nervous systems. Their receptors (PAC1, VPAC1, and VPAC2) are widely expressed in the brain but are also present in a multitude of peripheral target organs, including those of cardiovascular, renal, digestive, immune, endocrine, and reproductive systems. Due to cell-specific localization patterns, differing (VIP-ergic vs. PACAP-ergic) innervation configurations, and the existence of multiple alternate signaling pathways, the ligand/receptor interactions modulate physiological activities in highly specific manners (Vaudry et al., 2009). Importantly, these ligand receptor interactions, especially, PACAP/PAC1, are also known to modulate nervous system development, regeneration, and synaptic plasticity (Waschek, 1996, 2002, 2013; Otto et al., 2001; Hashimoto et al., 2006, 2011; Vaudry et al., 2009; Harmar et al., 2012; Rajbhandari et al., 2021). Moreover, we and others have investigated the involvement of PACAP in multiple behaviors in animals and in human disease by performing phenotype analyses of PACAP-deficient mice and genetic linkage analyses in humans. For example, PACAP-knockout mice exhibit increased locomotor activity, anxiety/anxolytic phenotypes, depression-like behavior, deficits in learning and memory, cognitive impairment (Hashimoto et al., 2001, 2009; Tanaka et al., 2006; Ishihama et al., 2010; Hattori et al., 2012; for reviews see: Hammack and May, 2015; Mustafa et al., 2015; Shibasaki et al., 2015; Farkas et al., 2017; Missig et al., 2017; Reglodi et al., 2018b; Johnson et al., 2020). Of note, the behavioral phenotype of PACAP-knockout mice varies under different environmental conditions and different genetic backgrounds (mouse strain), and with the specific gene sequences that were deleted in the different PACAP gene knockout models. In humans, the dysregulation of PACAP and the PAC1 receptor has been associated with schizophrenia, depression, and post-traumatic stress disorder (PTSD) (Hashimoto et al., 2007, 2010; Ressler et al., 2011; Mercer et al., 2016; Ross et al., 2020).

Recently, genetic studies aimed at the discovery of copy number variants (CNVs) have revealed that microduplications of *VIPR2* gene were strongly associated with schizophrenia (see below and Levinson et al., 2011; Vacic et al., 2011; Yuan et al., 2014; Li et al., 2016; Marshall et al., 2017). While both ligands (VIP and PACAP) appear to play roles in neural cell proliferation, maturation, synaptogenesis, protection and regeneration (see below and Falluel-Morel et al., 2007; Hill, 2007; Passemard et al., 2011), the pathological roles of PACAP and VIP and their mechanistic links to mental health disorders remain largely unknown. In the present review, we aimed to summarize physiological functions of the VPAC2 receptor, recent genetic research linking *VIPR2* duplications to schizophrenia, and relevant actions of VPAC2 receptors in animal and cell culture models.

## Localization and Physiological Functions of the VPAC2 Receptor

The VPAC2 receptor is a 7-transmembrane G-protein-coupled receptor (GPCR). Like PAC1 and VPAC1 receptors, VPAC2 couples with Gs-type trimeric G-proteins and activates adenylate cyclase, thereby producing cAMP and triggering the activation of protein kinase A (PKA). Additionally, the VPAC2 receptor activates phospholipase C (PLC) through both the pertussis toxin-sensitive (Gi/o) and -insensitive (Gq/11) pathways (Kim et al., 2000; MacKenzie et al., 2001). The VPAC2 receptor is widely distributed throughout the body in humans and is highly expressed in peripheral tissues such as the heart, stomach, pancreas, small intestine, thymus, prostate, testicle, ovary, and placenta. Activation of the VPAC2 receptor induces vasodilation, smooth muscle relaxation, and increases insulin secretion. In the brain, the VPAC2 receptor is highly expressed in neurons in the thalamus and hypothalamus, especially in the suprachiasmatic nuclei, as well as the cerebral cortex (Sheward et al., 1995; An et al., 2012). Its role regarding circadian rhythms has been studied extensively using VPAC2-deficient mice and VPAC2-overexpressing mice, demonstrating that the VPAC2 receptor is critically involved in the synchronization of neural activity and the regulation of firing frequency (Shen et al., 2000; Harmar et al., 2002; Cutler et al., 2003). Interestingly, we found that VPAC2-deficient mice exhibited normal fear learning, but a selective deficit in fear extinction (Ago et al., 2017). Notably, impaired extinction is a primary symptom of PTSD (Milad et al., 2009), a disease genetically linked to PACAP signaling (Ressler et al., 2011). Consistent with deficit in fear behavior in VPAC2 receptor knockout mice, altered synaptic structure in the prefrontal cortex was observed, with a decrease in numbers, lengths, and complexities of both apical and basal dendrites (Ago et al., 2017).

In addition to their presence in neurons, VPAC2 receptors are also expressed in astrocytes (Nishimoto et al., 2011) and oligodendrocytes (Lelievre et al., 2006), but not at detectable levels in microglia (Delgado et al., 2002). Only a few studies have addressed its regulation and physiological functions in glia. In a rat cortical cold injury model, VPAC2 receptors were found to be upregulated in cortical neurons and astrocytes in the penumbra (lesion border area), suggesting that VPAC2 receptors have an important function in brain trauma (Nishimoto et al., 2011). Furthermore, VIP immunoreactivity was detected in microglia within these lesions, a potential non-neuronal source of ligand. *In vitro* experiments utilizing cultured astrocytes showed that Ro 25-1553, a specific VPAC2 receptor agonist, induced in astrocytes morphological changes thought to mirror gliosis *in vivo.* Moreover, Ro 25-1553 up-regulated the glutamate transporters GLAST and GLT-1. These findings, along with, the unique spatiotemporal expression pattern of VPAC2 receptors in reactive astrocytes and VIP in nearby microglia, suggest that VIP–VPAC2 signaling may be involved in the induction of reactive astrogliosis and play an important role in neuroprotection against glutamate excitotoxicity. In another brain injury model, VIP and Ro 25-1553 were found to attenuate the ibotenic acid-induced cerebral white matter lesions in neonatal mice (Rangon et al., 2005). Moreover, the protective effect of VIP was absent in VPAC2-deficient mice. In other work, a VPAC2 receptor-specific agonist LBT-3627 exhibited neuroprotection against 1-methyl-4-phenyl-1,2,3,6-tetrahydropyridine (MPTP)-induced loss of dopaminergic neurons in a mouse model of Parkinson’s disease (Olson et al., 2015). LBT-3627 may have acted primarily via VPAC2 receptor on peripheral immune cells in this study because peripheral regulatory T cell function was enhanced in treated mice. Increased regulatory T cell activity may thus have dampened the inflammatory response in the brain, as evidenced by a reduction in microglia activation. Alternatively, or in addition, the VPAC2 agonist may have acted more directly within the brain, via receptors on neurons or astrocytes.

## Recent Genetic Studies on Schizophrenia

Schizophrenia is a severe mental health disorder, with a prevalence of about five cases per 1,000 individuals. The onset of this disease in men typically occurs in late teens to early twenties, and in women presents in the late twenties to early thirties. By the early 1990’s, concordance and heritability studies had already provided strong evidence that risk to the development of schizophrenia involves, and likely requires, multiple genetic alterations in concert with environmental factors (McGue and Gottesman, 1991). More recent molecular genetic approaches, including large-scale genome-wide association studies (GWAS) have led to the discovery of specific gene mutations and molecular pathways that confer risk to developing this disease. For example, a GWAS on schizophrenia that included approximately 37,000 patients and 113,000 healthy subjects revealed 108 genomic regions with a statistically significant association with schizophrenia (Schizophrenia Working Group of the Psychiatric Genomics Consortium, 2014). Additionally, recent large-scale studies employing deep RNA sequencing have successfully identified associated splicing quantitative trait loci (sQTL or splice QTL) (Battle et al., 2014; GTEx Consortium, 2015). Appling mRNA sequencing in the human pre-frontal cortex, a significant enrichment of sQTLs in schizophrenia risk loci was found (Takata et al., 2017). In particular, single nucleotide polymorphisms (SNPs) of sQTL that regulate the alternative splicing of genes such as *FXR1* and *SNAP91* were strongly associated with schizophrenia. *FXR1* encodes a homolog of fragile-X mental retardation protein (FMRP) that is responsible for fragile X syndrome. The encoded protein of *FXR1* is known to interact with FMRP (Zhang et al., 1995). Targets of FMRP are known to be involved in the genetic architectures of schizophrenia (Purcell et al., 2014). *SNAP91*, on the other hand, encodes the clathrin-associated protein AP180. AP180 is enriched in the presynaptic terminal of neurons and plays an essential role in synaptic neurotransmission (Koo et al., 2015). These findings suggest potential new genetic mechanisms by which brain sQTL SNPs regulate genes in which altered function results in enhance risk for schizophrenia.

Analysis of copy number variation (CNV) has become an important tool in the identification of novel and important mutations associated with disease and phenotypic traits. While CNVs typically explain only a small proportion of trait heritability, they may have large effects and functional consequences. Therefore, analyses of CNVs has a strong potential to lead to the elucidation of processes involved in pathogenesis of mental health disorders. For instance, it is well-known that 22q11.2 deletion presents a high risk for developmental neuropsychiatric disorders (odds ratio 20.3 and frequency 0.31% for schizophrenia) (Sullivan et al., 2012). Among other potentially relevant genes altered in this large multi-gene loci, a single nucleotide mutation of *RTN4R*, encoding the Nogo receptor 1, at 22q11.2 was significantly associated with schizophrenia. The *RTN4R* mutation results in decreased Nogo receptor binding with LINGO1, and thereby affects the formation of neuronal growth cones (Kimura et al., 2017).

These examples are meant to simply highlight the immense progress in the field of schizophrenia genetics that has occurred in the last decade. Readers are referred to excellent reviews in the literature that cover genetic advances in schizophrenia (for example, Avramopoulos, 2018; Dennison et al., 2020). With multiple technological advances, many types of genetic variants that increase the risk have been now recognized. While the type and contribution to the risk vary among genetic variants, there might be concordance in the functions of genes they implicate.

## VPAC2 Receptor Microduplication Linkage to Schizophrenia and Other Psychiatric Disease

In 2011, it was first reported that microduplications at 7q36.3 were strongly associated with schizophrenia (odds ratio 16.4 and frequency 0.24%) (Levinson et al., 2011; Vacic et al., 2011). All duplications overlapped with the *VIPR2* gene or were located within 89 kb upstream of the transcription start site. Increased *VIPR2* mRNA expression and cAMP accumulation in response to VIP and a VPAC2 receptor agonist BAY 55-9837 were observed in cultured lymphocytes of patients, demonstrating the functional significance of the microduplications (Vacic et al., 2011). These findings suggest the possible involvement of excessive signaling via the VPAC2 receptor in the pathophysiology of schizophrenia. Inheritances of the duplications at 7q36.3 were evaluated in three families and were shown to be *de novo* in one family, in which the duplication was observed in the proband but not in his/her unaffected parents. Subsequently, the association between the *VIPR2* CNV and schizophrenia were reported in a large scale study of the Han Chinese population (Yuan et al., 2014; Li et al., 2016). In another report, a novel smaller (35 kb) duplication upstream of the *VIPR2* promoter was discovered within a smaller cohort of about 300 schizophrenic patients in the Japanese population. This novel variant was relatively common (2%) in the Japanese population, but found not to be associated with increased schizophrenia risk (Aleksic et al., 2013). It can be concluded that this 35 kb variant does not capture the effects of the various gene segments identified by Vacic and colleagues, which together span a larger area of the *VIPR2* gene (Vacic et al., 2011).

Vacic et al. (2011) also showed that *VIPR2* CNVs exhibited higher frequencies in autism spectrum disorder (ASD) compared to control individuals. This requires confirmation because the number of autism-affected individuals was comparatively small. In other papers, the presence of a *VIPR2* CNV was reportedly present in an ASD patient and his ASD-afflicted father (Firouzabadi et al., 2017). In another type of work, neonatal blood concentrations of VIP, but not PACAP, were found to be higher in children later diagnosed with ASD compared to control subjects that did not (Nelson et al., 2001). In another genetic analysis, *VIPR2* SNPs were found to be significantly associated with depression (including bipolar disorder) (Soria et al., 2010) and hypomethylation at CpG sites of *VIPR2* was observed in DNA samples derived from the saliva of children with attention-deficit/hyperactivity disorder (Wilmot et al., 2016).

## Role of the VPAC2 Receptor in Synaptogenesis

Impairments of dendritic and synaptic density in pyramidal neurons across multiple brain regions, such as changes in dendritic arborization, dendritic spine number/type, and morphology, have been observed in schizophrenia (Moyer et al., 2015), indicating that altered synaptogenesis and/or plasticity may be an important factor underlying this mental health disorder. We determined more than two decades ago that VPAC2 gene expression in the postnatal mouse brain displays a pronounced peak at postnatal day 12, a time of active synaptogenesis, pruning and myelination, and then declines as animals reach adulthood (Waschek et al., 1996). In agreement with those findings, the Allen Mouse Brain Atlas reports a peak of VPAC2 gene expression at postnatal day 14, with highest levels in prosomeres 2 and 3 (putatively corresponding to the developing hypothalamus and thalamus), as well as scattered signals in the cortex, including the prefrontal cortex. These observations suggest that VPAC2 receptors might play an important role in postnatal maturation of the nervous system, and that increased, improperly timed, or ectopic activation might impair the establishment of circuits involved in cognition.

We thus used pharmacological approaches to determine anatomical and behavioral changes that might occur due to overactive VPAC2 signaling during the early postnatal period (Ago et al., 2015). We subcutaneously administered Ro 25-1553 once daily over the first 2 weeks of life. Analysis of synaptic proteins levels by Western blot at postnatal day 21 revealed significant reductions of synaptophysin and postsynaptic density protein 95 (PSD-95) in the prefrontal cortex, but not in the hippocampus (Ago et al., 2015). The same postnatally restricted treatment resulted in a long-term disruption of prepulse inhibition of the acoustic startle in adult (3–4 month old) mice. On the other hand, no effects were observed in locomotor activity in the open-field test, sociability in the three-chambered social interaction test, or memory function in the fear conditioning or extinction (Ago et al., 2015). In another study, we found that VPAC2 receptor-deficient mice exhibited abnormal dendritic morphology of the prefrontal cortex neurons, but not basolateral amygdala neurons (Ago et al., 2017).

Recently, Tian et al. (2019) developed a conditional human *VIPR2* CNV bacterial artificial chromosome (BAC) transgenic (h*VIPR2*-BAC tg) mouse model of *VIPR2* CNV. They reported that h*VIPR2*-BAC tg mice showed cognitive, sensorimotor gating, and social behavioral deficits and decrease in the complexity of dendritic arborization of the striatal spiny projection neurons. These findings suggest that the VPAC2 receptor plays an important role in the regulation of the dendritic morphology and that the *VIPR2*-linkage to mental health disorders may be due in part to overactive VPAC2 receptor signaling during a critical time of synaptic maturation in the prefrontal cortex and striatum circuitry. A limitation of these mouse genetic models, however, is that gene function is impaired during development as well as in the adult. Because VPAC2 expression is altered in both time periods, effects on behavior cannot necessarily be attributed to alterations occurring development. Furthermore, compensatory mechanism might come into play with long term alterations in gene expression. Thus pharmacological approaches have been used to study specific roles of VPAC2 receptors in brain development.

## Effects of VPAC2 Receptor Activation on Axon and Dendrite Growth in Cultured Cells

**FIGURE 1:**
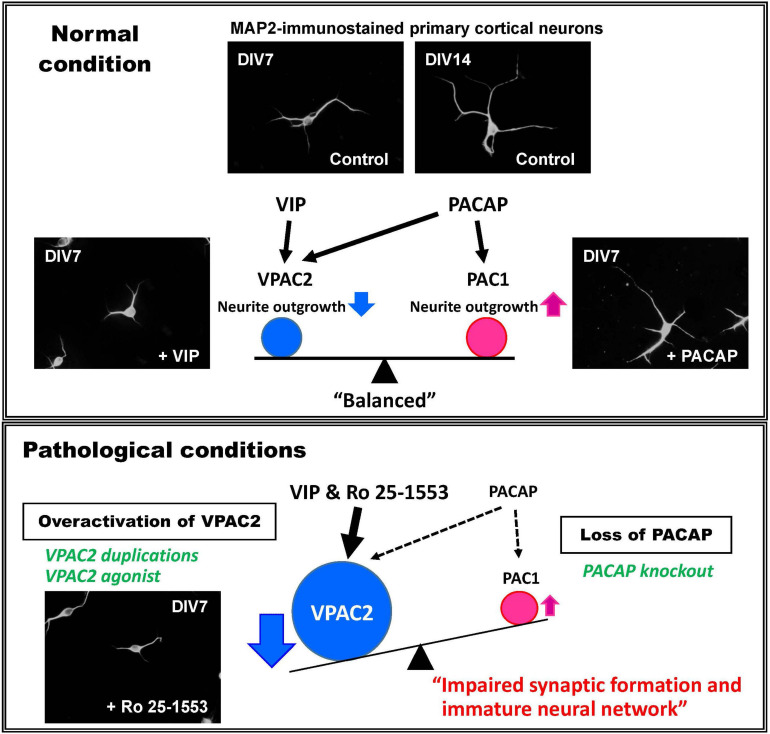
Proposed mechanism for the roles of VIP and PACAP system in neural development and mental health disorders. Mouse primary embryonic cortical neurons extend axons and dendrites *in vitro* in the absence of added peptide. Addition of VIP results in a reduction in total numbers and lengths of neuronal dendrites via the VPAC2 receptor, whereas PACAP selectively facilitates the elongation of dendrites via the PAC1 receptor (Takeuchi et al., 2020). To explain these differential effects, it is proposed that VPAC2 and PAC1 signaling undergoes differential timed activations in brain development under normal (physiological) conditions. When the VPAC2 receptor activity is enhanced by *VIPR2* duplications or by pharmacological activation, or if PACAP–PAC1 signaling is reduced by PACAP deficiency, the VPAC2 signaling would be expected to become relatively amplified. This might cause the delay of neural maturation and thus impaired synaptic function, leading to brain dysfunction.

Given that altered dendritic arborizations were observed in the medial prefrontal cortex of VPAC2 receptor knockout and VPAC2 receptor agonist-treated mice, we have studied the ability of native PACAP and VIP and Ro 25-1553 to regulate the formation of axons and dendrites *in vitro* using cultured neurons from wild-type embryonic day 16 mouse cortices. VIP and Ro 25-1553 were found to dose-dependently inhibit the growth of both axons and dendrites in these cultures, effects that were fully blocked by H89, a PKA inhibitor and a VPAC2 receptor antagonist PG 99-465 (Takeuchi et al., 2020). Interestingly, PACAP had no detectable effect on axons or dendrites at lower doses, but stimulated growth at relatively high doses, an effect which was blocked by a MAPK/ERK kinase (MEK) inhibitor U0126 (and not H89). Our interpretation of this data is that VPAC2 and PAC1 receptor activation have opposing effects on developing axons and dendrites, where VPAC2 inhibits and PAC1 stimulates growth. The stimulatory effects on PAC1 by lower concentrations of PACAP in this cell culture model are canceled by PACAP’s inhibitory action on VPAC2 receptors. VIP, on the other hand, only inhibits growth because it does not significantly activate PAC1 receptors. In physiological settings, the ultimate effects depend on the balance of both ligands and receptors (Figure 1). Notably, PAC1 receptors are expressed as early as embryonic day 9.5 and remain expressed thereafter (Shioda et al., 1997; Waschek et al., 1998), whereas VPAC2 receptors do not become strongly expressed until about postnatal day 6 to day 12. Thus, during early development the presence of PAC1 receptors positively drives axon and dendrite growth. The later onset of VPAC2 receptor expression may then provide a regulatory mechanism to control this process. Collectively, we propose that miss-timed, ectopic, or lack of VPAC2 receptor during critical times of postnatal development will result in a failure to fine tune synapse formation, resulting in synaptic aberrations and the failure to properly develop circuits that are critical to cognition. Moreover, we propose that similar processes are affected in adult mice in the context of synaptic plasticity.

## Conclusion

In this review, we present an overview of human genetic studies implicating *VIPR2* CNVs as a risk factor for developing schizophrenia, along basic research findings in mice and cell culture models that provide potential mechanisms to explain the linkage. Our studies suggest that excessive activation of VPAC2 signaling during development influences the formation and maturation of neural structures in the brain such as the prefrontal cortex, thereby impairing sensory information processing and cognitive function. Reported *VIPR2* duplications directly lead to excessive signaling via the VPAC2 receptor owing to increased VPAC2 expression. In addition, considering that the PAC1 receptor is selectively activated by PACAP, VIP–VPAC2 signaling may become amplified under certain conditions of PACAP deficiency such as aging (Reglodi et al., 2018a). A detailed analysis for abnormalities in development, maturation and tuning of neurons due to VPAC2 receptor activation may help to uncover the molecular mechanisms underlying the etiology of mental health disorders such as schizophrenia and ASD.

## Author Contributions

YA, SA, HH, and JW wrote the manuscript. YA and JW reviewed and approved the manuscript and held all the responsibilities related to this manuscript. All authors reviewed and approved the manuscript.

## Conflict of Interest

The authors declare that the research was conducted in the absence of any commercial or financial relationships that could be construed as a potential conflict of interest.
